# Dysregulation of Different Modes of Programmed Cell Death in Rheumatoid Arthritis Fibroblast‐Like Synoviocyte

**DOI:** 10.1111/1756-185x.70445

**Published:** 2025-10-23

**Authors:** Xiaorong Zhi, Hong Zhu, Xiaoyan Sun, Zhaoxia Wang, Lin Wang, Guanhua Song

**Affiliations:** ^1^ School of Clinical and Basic Medical Sciences Shandong First Medical University & Shandong Academy of Medical Sciences Jinan China; ^2^ Shandong Provincial Key Laboratory for Rheumatic Disease and Translational Medicine The First Affiliated Hospital of Shandong First Medical University & Shandong Provincial Qianfoshan Hospital Jinan China; ^3^ Biomedical Sciences College & Shandong Medicinal Biotechnology Centre Shandong First Medical University & Shandong Academy of Medical Sciences Jinan China

**Keywords:** apoptosis, cell death, rheumatoid arthritis, synoviocytes, therapeutics

## Abstract

Rheumatoid arthritis (RA) is a chronic inflammatory disease characterized by chronic synovitis and skeletal joint deformities, often accompanied by systemic symptoms. Over the past few decades, various susceptibility factors for RA have been revealed, and numerous therapeutic drugs have been developed, including analgesics, glucocorticoids, non‐steroidal anti‐inflammatory drugs (NSAIDs), disease‐modifying antirheumatic drugs (DMARDs), and biological agents (bDMARDs). Despite the availability of multiple treatment options, the therapeutic outcomes for some patients remain suboptimal due to the complex pathogenesis of RA. As a key pathological mechanism, programmed cell death (PCD) in RA has received extensive attention. Dysregulation of PCD in RA impacts the progression of the disease. This article systematically reviews the roles of various cell death modalities, including apoptosis, necroptosis, ferroptosis, pyroptosis, and autophagy in the pathophysiology of RA, aiming to provide a theoretical basis and direction for the discovery of new therapeutic targets and drug development.

## 1 Introduction

Rheumatoid arthritis (RA) is a chronic autoimmune disease characterized by chronic inflammation of the joint synovium and destruction of joint structures. Clinically, it presents with symmetric pain, stiffness, and swelling in one or more joints, which may eventually lead to joint destruction, functional impairment, and even disability [1]. The pathological features of RA include proliferation of synovial cells, neovascularization, inflammatory cell infiltration, and destruction of cartilage and bone tissues [2]. The main drugs currently used to treat RA include non‐steroidal anti‐inflammatory drugs (NSAIDs), glucocorticoids (GCs), and disease‐modifying antirheumatic drugs (DMARDs) such as methotrexate (MTX) [3, 4]. However, these drugs only alleviate symptoms or slow disease progression, are costly, and have numerous adverse effects. Consequently, developing new therapeutic strategies has become a priority [5, 6].

Therefore, a thorough exploration of the specific roles and regulatory mechanisms of these cell death modalities in RA can not only enhance our understanding of the pathogenesis of RA but also provide important clues for the development of new therapeutic strategies. This article aims to systematically review the dysregulation of key programmed cell death (PCD) modalities in RA, including apoptosis, necroptosis, pyroptosis, ferroptosis, and autophagy. It analyzes their interactions and effects on the survival of synovial fibroblasts (FLS), while also evaluating the potential of novel therapeutic strategies targeting PCD pathways. Furthermore, it proposes future research directions to provide a theoretical basis for precision treatment of RA.

## 2 The Role of FLS in RA

FLS play a crucial role in both the healthy and diseased states of joint synovium. In normal joints, FLS contribute to joint health by synthesizing synovial fluid and regulating the composition of the extracellular matrix, providing lubrication and nutrition to the joint cartilage [7, 8]. However, during the progression of RA, the behavior of FLS changes significantly; the resistance of FLS cells to cell death leads to excessive proliferation of synovial cells, causing pathological hyperplasia of the joint synovium and further joint destruction [9]. Concurrently, the excessive secretion of pro‐inflammatory cytokines, angiogenic factors, and matrix metalloproteinases (MMPs) by FLS can contribute to tissue infiltration and destruction, further triggering RA progression [10]. FLS also secrete various chemokines to recruit macrophages, T cells, and B cells to the joints, enhancing the inflammatory response and indirectly damaging bones and joints. Moreover, FLS can inhibit the apoptosis of T cells and B cells, prolonging the inflammatory response and intensifying damage to bones and joints [11].

In summary, dysfunction, especially resistance to apoptosis of FLS, can lead to abnormal synovial proliferation, inflammatory cell infiltration, cartilage and bone erosion, joint destruction, and deformities, ultimately resulting in RA progression.

## 3 FLS Apoptotic Dysregulation

**FIGURE 1:**
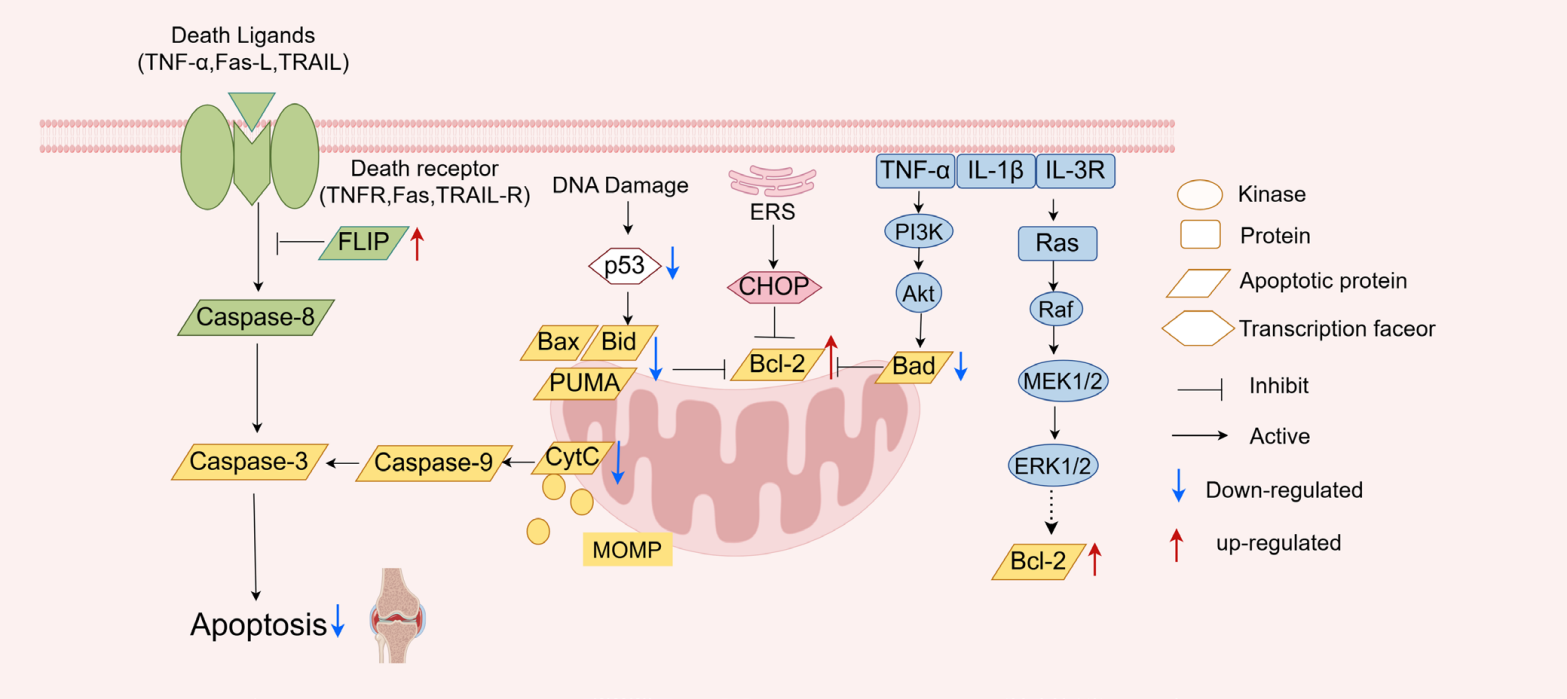
Coordinated anti‐apoptotic network in RA FLS and cross‐pathway interfaces. In RA FLS, a complex anti‐apoptotic network operates through cross‐pathway interactions: PI3K/Akt signaling upregulates Bcl‐2/Bcl‐xL expression and phosphorylates pro‐apoptotic proteins (eg., Bad), stabilizing mitochondrial outer membrane integrity and suppressing caspase cascades (eg., caspase‐9 → caspase‐3). p53 promotes mitochondrial apoptosis by transcriptionally activating Bax/PUMA/Noxa while repressing SLC7A11, depleting GSH and weakening GPX4 activity. This dual role links apoptosis to ferroptosis via lipid peroxidation. Caspase‐8 serves as a master switch: its activation triggers caspase‐3/7‐mediated apoptosis, while its inhibition permits RIPK1/RIPK3‐MLKL‐driven necroptosis. Caspase‐8 and caspase‐3 can also process gasdermins, influencing transitions between pyroptosis and apoptosis. ERS (eg., PERK/IRE1α/ATF6) and NF‐κB/PI3K‐Akt signaling converge to enhance survival programs, lower apoptotic sensitivity, and modulate pyroptosis/ferroptosis thresholds. This coordinated network highlights the interplay among survival signals, mitochondrial integrity, and cell death regulation, explaining the abnormal survival and proliferation of RA FLS under pathological conditions. The main figure was drawn by Figdraw.

One of the key mechanisms involved in the pathogenesis of RA is the dysregulated apoptosis, leading to abnormal proliferation and decreased apoptosis of FLS [12]. Apoptosis is a fundamental mechanism for organism development, tissue remodeling, and immune regulation. However, in the RA environment, FLS exhibit a significant imbalance in apoptosis [10]. Specifically, FLS regulate Bcl‐2 family proteins, inhibit Caspase activity, inactivate p53 function, and activate anti‐apoptotic signaling pathways (such as PI3K/Akt, MAPK, NF‐κB), thereby constructing a complex anti‐apoptotic network [13]. In addition, the endoplasmic reticulum stress (ERS) tolerance mechanism also provides FLS with a survival advantage in the harsh microenvironment, further exacerbating their resistance to apoptosis [14]. The following sections will discuss the molecular mechanisms related to apoptosis imbalance in FLS during RA progression (Figure 1).

### 3.1 The Role of p53

In RA, p53, a crucial tumor suppressor and transcription factor, plays a significant role in regulating apoptosis [15]. Under normal conditions, p53 activates pro‐apoptotic factors in response to DNA damage and cellular stress, triggering the apoptotic process. However, in RA, p53 is frequently inactivated or mutated, leading to apoptosis resistance in FLS. In the RA microenvironment, inflammatory cytokines such as TNF‐α and IL‐6 inhibit p53 activity, further enhancing FLS resistance to apoptosis signals [16].

The downstream signaling of p53 is also disrupted in RA. For example, p53‐induced pro‐apoptotic genes such as Bax, PUMA, and Noxa show reduced expression in RA FLS, which diminishes the sensitivity of cells to apoptotic signals [17]. Moreover, p53 is involved in autophagy regulation, which enhances FLS survival and further contributes to their abnormal proliferation and apoptosis resistance in RA [18]. p53 also plays a role in ferroptosis regulation, promoting lipid peroxidation and FLS death by inhibiting SLC7A11 expression [19]. However, certain p53 mutations (eg., p53^3KR) and the p53‐p21‐DPP4 axis may counteract ferroptosis by maintaining glutathione (GSH) homeostasis, forming a complex survival network [20].

In summary, the functional loss and mutations of p53 in RA not only disrupt the apoptosis signaling pathway but also enhance FLS survival and proliferation through mechanisms like autophagy and ferroptosis regulation. These changes contribute to the pathogenesis of joint inflammation and destruction in RA, highlighting p53 as a potential therapeutic target for reversing FLS apoptosis resistance.

### 3.2 ERS Regulation of RA Apoptosis

ERS plays a critical role in RA. When cells encounter stimuli (such as protein misfolding or Ca^2+^ imbalance), ERS is regulated through the unfolded protein response (UPR), activating sensors like IRE1α, PERK, and ATF6α to restore cellular homeostasis [14]. In early stages, UPR helps the cell adapt to stress, but prolonged stress leads to pro‐apoptotic signaling. In RA, synovial tissue shows high expression of key ERS proteins (BiP, XBP1s, PERK), indicating a close relationship between ERS, inflammation, and oxidative stress. The mechanisms underlying ERS‐related apoptosis resistance may involve abnormal regulation of CHOP/Bcl‐2 imbalance, disrupted calcium signaling (eg., calpain activation), and interference of pro‐apoptotic UPR branches by the inflammatory microenvironment [21]. Targeting ERS pathways may represent a new strategy to overcome FLS apoptosis resistance [22].

In summary, FLS resist apoptosis through multiple mechanisms, which play an important role in the pathogenesis of RA. In‐depth study of these mechanisms will help reveal the pathophysiology of RA and provide potential targets for the development of new therapeutic strategies.

## 4 The Dysregulation of Other PCDs in FLS

PCD is a controlled death process involving complex signaling cascades, coordinated by a series of specific effector molecules, ensuring precise execution of cell death [23]. Besides, apoptosis plays a significant role in RA; current researchers indicate PCD, such as necroptosis, pyroptosis, ferroptosis, NETosis, autophagy, and cuproptosis may exacerbate inflammatory responses [24]. These complex forms of cell death collectively contribute to the pathology of RA. Targeting PCD may offer potential therapeutic avenues for RA, warranting further research and development. Next, we will delve into specific cell death modalities and their roles in RA to better understand how these death mechanisms influence disease progression and treatment strategies (a summary of key inhibitors is presented in Table 1).

**TABLE 1:** Dysregulated programmed cell death pathways in rheumatoid arthritis.

PCD pathway	Key features	Role in RA	The therapeutic targets and inhibitors
Necroptosis	Programmed cell death mediated by RIPK1, RIPK3, and MLKL involves cell swelling, membrane rupture, and the release of DAMPs and inflammatory factors	TNF‐α activates necroptosis in RA FLS, leading to upregulation of RIPK3 expression, which drives the release of inflammatory factors (IL‐1β, IL‐6) and exacerbates arthritis inflammation and synovial hyperplasia	Targets: RIPK1, RIPK3, MLKL; Inhibitors: RIPK1 inhibitors (KW2449, Necrostatin‐1), RIPK3 inhibitors (GSK'872, AZ‐628, etc.), MLKL inhibitors (NSA, GW806742X)
Pyroptosis	Activation of inflammasomes (such as NLRP3) induces the activation of caspase‐1/11, which cleaves GSDMD to form membrane pores, leading to cell swelling and the release of IL‐1β/IL‐18	In RA synovial tissue, abnormal activation of NLRP3 inflammasomes releases IL‐1β and IL‐18, mediating pyroptosis of FLS and immune cells, exacerbating joint inflammation and cartilage destruction	Targets: NLRP3, GSDMD, Caspase‐1; Inhibitors: NLRP3 inhibitors (MCC950, CY‐09), GSDMD inhibitors (Disulfiram, LDC7559)
Ferroptosis	Lipid peroxidation is driven by iron ions and GPX4 inhibition, characterized by mitochondrial shrinkage, increased membrane density, and loss of cristae	In RA, oxidative stress and iron overload promote ferroptosis, aggravating RA synovial inflammation and joint damage. High expression of SLC7A11 in FLS confers resistance to ferroptosis, enhancing invasiveness	Targets: GPX4, SLC7A11; Direct inhibitors: Icariin; Indirect regulation: Coenzyme Q10, Resveratrol, Quercetin, Curcumin, and other natural antioxidants
NETosis	NETs (Neutrophil Extracellular Traps) are mediated by DNA, histones, and proteases (such as MPO, NE)	NETs accumulate in RA synovial tissue, releasing pro‐inflammatory factors that exacerbate RA autoimmune responses, promoting synovitis and cartilage degradation	Targets: PAD4, MPO; Inhibitors: PAD4 inhibitors (Cl‐amidine), DNase I, NOX inhibitors (DPI)
Autophagy	Damaged organelles and proteins are cleared via the autophagosome‐lysosome pathway to maintain homeostasis. Overactivation of this process can lead to cell death. Core molecules include LC3, Beclin‐1, and mTOR pathway regulation	In RA synovial tissue, autophagy‐related protein expression is upregulated, promoting FLS survival and invasiveness. Autophagy deficiency leads to mitochondrial damage, promoting necroptosis, apoptosis, and oxidative stress	Targets: mTOR, Beclin‐1, ATG5, LC3; Inhibitors: Autophagy activators (Rapamycin), Autophagy inhibitors (Chloroquine), PI3K inhibitors (3‐MA)
Cuprotosis	Copper ion accumulation causes mitochondrial damage through FDX1‐mediated inhibition of mitochondrial respiratory chain complex IV, leading to oxidative stress	In RA, serum copper levels are elevated and correlated with disease activity. FLS exhibit copper death inhibition due to glutamine consumption, exacerbating abnormal cell proliferation and resistance to apoptosis	Targets: Positive regulators of copper death (FDX1, DLAT) and negative regulators (MTF1, GLS); Inhibitors: Copper chelators (such as Penicillamine)

*Note:* The table summarizes key features, pathogenic roles in RA synovium, and potential therapeutic targets/inhibitors for six PCD modalities: necroptosis, pyroptosis, ferroptosis, NETosis, autophagy‐related death, and cuproptosis.

Abbreviations: DAMPs, damage‐associated molecular patterns; FLS, fibroblast‐like synoviocytes; IL, interleukin; NETs, neutrophil extracellular traps.

### 4.1 Necroptosis

Necroptosis is mainly initiated by members of the tumor necrosis factor receptor (TNFR) and Toll‐like receptor (TLR) families, interferon, intracellular RNA and DNA sensors, and other mediators [25, 26, 27]. Subsequently, the protein kinase RIPK1 (receptor‐interacting protein kinase 1) and RIPK3 interact with the receptor protein, which transduces death signals and further recruits and phosphorylates MLKL (mixed lineage kinase domain‐like protein), forming a specific signaling complex known as the “necrosome,” a process essential for necroptosis [28].

In RA, necroptosis promotes joint inflammation. Studies have shown that plasma levels of RIPK1 and MLKL are higher in RA patients than in healthy individuals, with these levels positively correlating with the severity of RA, suggesting that RIPK1 and MLKL may serve as novel biomarkers for RA [29]. Additionally, it has been reported that the absence of IFN‐γ enhances the expression of RIPK1, RIPK3, and MLKL in collagen‐induced arthritis (CIA) mice, increases the number of Th17 cells, and promotes the release of IL‐17 and TNF‐α, thus exacerbating inflammation and joint damage in RA [30]. Necroptosis also drives chondrocyte death and aggravates cartilage degeneration and inflammation.

Studies have found that small molecule inhibitors targeting RIPK1 hold potential as therapeutic strategies for RA. For example, KW2449, by inhibiting RIPK1‐dependent necroptosis, was used in adjuvant arthritis (AA) rat cartilage; expression levels of RIPK1, RIPK3, and P‐MLKL mediated by ASIC‐1a were elevated, correlating with necroptosis [31]. The RIPK1 inhibitor Necrostatin‐1 can alleviate joint damage and inflammation. Moreover, RIPK3 inhibitors (such as GSK'872 and AZ‐628) and MLKL inhibitors (such as NSA and GW806742X) have shown clear potential in suppressing cartilage degeneration and inflammation. Future studies should focus on optimizing inhibitor specificity, exploring multi‐target combination strategies, and validating their long‐term safety and efficacy in humans.

### 4.2 Pyroptosis

Pyroptosis is a pro‐inflammatory form of PCD that morphologically differs from apoptosis and necrosis. The main features include cell swelling, nuclear condensation, and the formation of bubble‐like large vesicles on the cell membrane, ultimately leading to membrane rupture [32, 33, 34]. Pyroptosis is triggered by inflammasome activation, which is designed to defend against harmful pathogens and maintain internal homeostasis. Meanwhile, excessive activation of the NLRP3 inflammasome can lead to the release of inflammatory mediators, potentially resulting in inflammatory responses and tissue damage [35, 36, 37].

Studies have shown that pyroptosis is observed in FLS of RA patients. High levels of GSDMD (a key molecule in pyroptosis), and NLRP3, caspase‐1, IL‐18, IL‐6, and IL‐1β (key molecules in inflammasome), were detected in their serum and synovial tissues [38, 39]. These findings suggest that pyroptosis is involved not only in the pathogenesis of RA but also that its incidence may be positively correlated with RA activity. Therefore, inhibiting core molecules of pyroptosis may become a key strategy to prevent the progression of RA.

Currently, several inhibitors have been developed to regulate NLRP3 inflammasomes and GSDMD. MCC950, a specific NLRP3 inhibitor, directly interacts with the NACHT domain of NLRP3, blocking ASC oligomerization and significantly suppressing synovitis and cartilage erosion. CY‐09, by interacting with the Walker A motif of NLRP3, prevents ATP binding and activation of NLRP3 [40]. Additionally, GSDMD, a downstream effector protein of the NLRP3 inflammasome, has led to new hope for RA treatment with small molecule inhibitors such as disulfiram and LDC7559. In traditional Chinese medicine research, Baihu Guizhi Decoction (BHGZD) inhibits NLRP3 inflammasome activation and pyroptosis; quercetin targets CASP1 and regulates pyroptosis, effectively alleviating the immune‐inflammatory imbalance in RA [41, 42]. Moreover, Salicylalazine (SAZ) can exert significant anti‐arthritic effects by regulating the TLR4/NLRP3/GSDMD signaling axis‐mediated pyroptosis cascade. Inhibition of ASIC1a function significantly reduces pyroptosis in RA FLS [43]. Notably, Pentraxin 3 (PTX3) has emerged as a novel biomarker for RA diagnosis. After binding to complement C1q, PTX3 can induce monocytic pyroptosis, releasing large amounts of IL‐1β, IL‐6, and other inflammatory factors, creating an “inflammatory cytokine storm” that exacerbates joint destruction [44].

### 4.3 Ferroptosis

Ferroptosis is an iron‐dependent and lipid peroxidation‐driven form of PCD characterized by excessive iron accumulation, increased iron‐dependent ROS, and lipid peroxidation, leading to membrane rupture, release of contents, and necrosis‐like cell death [45, 46]. Ferroptosis releases inflammatory cytokines such as IL‐1β, TNF‐α, and IL‐6, triggering an inflammatory response.

Studies have shown higher iron content and increased levels of ROS and lipid peroxidation in the serum and synovial fluid of RA patients, with abnormal antioxidant systems [47, 48]. ROS levels are positively correlated with RA disease severity scores and are one of the key factors in disease progression [49]. Elevated lipid ROS levels, which are regulated by GPX4, can inhibit ferroptosis. Notably, ROS continuously stimulate TNF‐α production by activating the NF‐κB pathway, forming a positive feedback loop of ROS/TNF‐α [50]. The increased iron intake accelerates the vicious cycle of inflammation and hemorrhage, leading to synovial hyperplasia and invasion of joint cartilage.

Recent studies indicate that glycine drives ferroptosis in RA FLS by methylating the S‐adenosylmethionine (SAM)‐related GPX4 promoter and enhancing this effect [51]. This discovery provides crucial clues for the molecular mechanisms of ferroptosis in RA and the development of new therapeutic strategies. Moreover, the bioactive peptide G1dP3 promotes ferroptosis in RA FLS through the p53/SLC7A11 axis, demonstrating its therapeutic potential in RA. Similarly, drugs like sulfasalazine regulate ferroptosis by inhibiting the Xc‐ system and the expression of GSH and GPX4. On the other hand, it also induces ferroptosis by increasing iron ion levels and generating excessive lipid ROS [52]. Natural antioxidants also play a role in ferroptosis regulation. For example, Icariin inhibits ferroptosis in synovial cells through activation of the Xc‐/GPX4 axis and exerts a protective effect [53].

### 4.4 Netotic Cell Death

Netotic cell death is characterized by the release of extracellular net‐like DNA‐protein structures known as neutrophil extracellular traps (NETs) [54]. NETs consist of DNA, histones, neutrophil‐specific alarm proteins, myeloperoxidase (MPO), neutrophil elastase (NE), and cathepsin G, and their function is to eliminate harmful pathogens [55]. Neutrophils, under various signaling stimuli, generate NETs, which ultimately lead to cell death and the release of NETs. This process is referred to as NETosis.

NETosis serves as an inflammation‐driving factor in RA [56]. In RA patients, activated neutrophils in the synovial fluid significantly increase, participating in the synergistic effects of immune complexes and the complement system, leading to excessive NETosis, the release of inflammatory mediators, and causing inflammation and joint destruction [57]. The spontaneous increase in neutrophil populations is associated with ROS production, MPO expression, and PAD4‐mediated citrullination, and NETosis derivatives are closely correlated with the severity of RA [57].

Therefore, targeting NETosis has been considered a new therapeutic strategy in RA treatment. Suppressing key molecules of NETosis, such as PAD enzyme inhibitors to inhibit PAD2/PAD4 activity, reduces histone citrullination and NET formation [58, 59]. Furthermore, small molecule inhibitors like Cl‐amidine have been validated in animal models for their anti‐inflammatory effects, and NOX inhibitors such as diphenyl iodonium (DPI) and protease inhibitors help reduce NET release and tissue damage factors [60].

Regarding natural compounds and traditional drugs, studies have shown that emodin, by inhibiting autophagy and NETosis, alleviates RA symptoms in the AA model [61]. Other potential drugs, such as hydroxychloroquine and MTX, may indirectly influence NETosis by regulating immune cell functions. Furthermore, anti‐TNF‐α and anti‐IL‐6R treatments can reduce NET formation and alleviate inflammation. Notably, NETosis‐derived markers such as serum MPO‐DNA complexes and calprotectin levels are positively correlated with anti‐citrullinated protein antibodies (ACPA), rheumatoid factor (RF) titers, and disease severity, and could serve as biomarkers for RA diagnosis and therapeutic efficacy evaluation [62]. In conclusion, excessive NETosis promotes RA progression by facilitating immune complex formation and the release of inflammatory mediators. Targeting NETosis can effectively alleviate inflammation, reduce NET formation, and slow the pathological progression of RA.

### 4.5 Autophagy

Autophagy is a non‐apoptotic form of PCD that primarily removes damaged organelles and waste proteins via the autophagosome‐lysosome pathway, maintaining cellular homeostasis [63, 64]. However, it can sometimes have adverse effects by disrupting the balance between life and death by selectively degrading proteins associated with other types of PCD [65].

In RA, autophagy exhibits complex roles. Analysis of synovial tissue from RA patients shows increased expression of autophagy‐related proteins (such as Beclin1, ATG5, LC3), which are positively correlated with inflammatory markers and autoantibody levels [66]. Autophagy is closely related to the development of RA, and autophagy inhibition has become a new therapeutic strategy for improving RA.

Regulating the autophagy process has become a new direction in RA treatment. For example, triptolide can block the CaMKKβ‐AMPK‐mTOR pathway and inhibit FLS autophagy, while tomorutin extract can downregulate LC3B protein, upregulate caspase‐3, and correct the abnormal expression of ULK‐1, thus alleviating synovial inflammation and bone erosion [67, 68]. Additionally, intervention in the AKT/PI3K signaling axis, such as with dexamethasone, astragalus polysaccharides, and hesperidin/5‐methoxyflavone, targets AKT signaling to inhibit autophagy in synovial cells. Moreover, autophagy inhibitors like 3‐MA/chloroquine can directly inhibit autophagosome formation and reduce the levels of inflammatory factors [69].

### 4.6 Cuproptosis

Cuproptosis is a novel form of cell death triggered by copper ion overload, independent of apoptosis, necroptosis, pyroptosis, and ferroptosis. Copper, as an essential trace element, is a cofactor for several enzymes, such as cytochrome oxidase (complex IV of the respiratory chain) and superoxide dismutase (SOD), playing a crucial role in maintaining normal cellular functions. However, excessive copper can lead to toxicity, triggering the cuproptosis mechanism [70].

In cuproptosis, copper overload primarily induces cell death through FDX1/DLAT‐mediated mitochondrial dysfunction [71]. The specific mechanism involves ferredoxin 1 (FDX1), a core regulator of cuproptosis, whose activity is directly modulated by excessive copper ions [72]. FDX1 reduces Cu^2+^ to Cu^+^, enhancing the reactivity of copper ions. Activated FDX1 further catalyzes excessive modification of mitochondrial lipoylated proteins, such as DLAT. As a key component of the pyruvate dehydrogenase (PDH) complex, abnormal lipoylation of DLAT leads to protein aggregation and disrupts mitochondrial protein homeostasis. Lipoylation modification of DLAT induces protein aggregation, impairs the function of iron–sulfur cluster proteins (Fe‐S proteins), and causes inactivation of the tricarboxylic acid (TCA) cycle and respiratory chain complexes. Ultimately, this results in the collapse of mitochondrial membrane potential and accumulation of reactive oxygen species (ROS), triggering cuproptosis.

Studies have shown that copper levels in the serum of RA patients are significantly elevated and closely related to disease activity. Specifically, in patients with active RA, copper levels are positively correlated with erythrocyte sedimentation rate (ESR) and negatively correlated with hemoglobin levels [73]. Additionally, the overall levels of glucose and glutamine in RA FLS are reduced, indicating enhanced consumption, suggesting that glutamine plays a critical role in FLS proliferation [12].

Mechanistically, glutamine fuels the TCA cycle via GLS‐mediated conversion to glutamate and α‐ketoglutarate (α‐KG) [74]. Adequate TCA flux maintains mitochondrial lipoylation dynamics of pivotal E2 components such as DLAT, which is required for FDX1‐dependent cuproptosis execution [75]. Under glutamine depletion, reduced α‐KG supply diminishes TCA flux, lessens DLAT lipoylation substrates, and attenuates copper‐induced aggregation of lipoylated proteins, thereby elevating the death threshold and suppressing cuproptosis. In RA‐FLS, which display glutamine addiction to sustain proliferation and invasion, nutrient competition and metabolic reprogramming under inflammatory stress can paradoxically blunt cuproptosis and favor synovial hyperplasia [76]. Functionally, restoring anaplerosis (eg., α‐KG supplementation or GLS modulation) or leveraging copper ionophores under controlled metabolic support may re‐sensitize RA FLS to cuproptosis and restrain pannus overgrowth.

Currently, therapeutic strategies targeting cuproptosis in RA involve regulating copper homeostasis by intervening with drugs (such as D‐penicillamine and other copper chelators) to modulate copper absorption, distribution, and excretion, restoring copper homeostasis [77]. Alternatively, targeting copper ion transporters to develop synovial‐targeted copper ion carriers (eg., coupling FLS‐specific antibodies) can help reduce inflammation [78]. Furthermore, targeting cuproptosis‐related genes, including positive regulators of cuproptosis (such as FDX1, DLAT) and negative regulators (such as MTF1, GLS), by developing gene therapy or small molecule inhibitors, could potentially modulate copper death sensitivity and improve RA symptoms [70]. Combination therapies, using copper homeostasis regulators together with traditional anti‐rheumatic drugs (such as DMARDs and biologics), may enhance efficacy and reduce side effects [78].

## 5 Interactions Between Cell Death Pathways

The signaling pathways involved in apoptosis, necroptosis, pyroptosis, ferroptosis, NETosis, and autophagy are distinct, yet there are tight interconnections and regulatory mechanisms that form a “crosstalk” network, collectively contributing to the decision‐making process in regulating cell fate. Therefore, exploring the interplay and synergy of these cell death pathways is crucial for understanding disease progression and developing new therapeutic strategies.

### 5.1 Interaction Between Autophagy and Apoptosis

In RA, there is a significant negative correlation between autophagy and apoptosis; inducing autophagy may lead to self‐protective apoptosis resistance in RA cells. Studies show that autophagy markers (Beclin‐1, LC3B) are significantly upregulated in synovial tissues of RA patients, while apoptosis levels are notably reduced, suggesting a negative correlation between the two. Inhibition of autophagy can reverse the anti‐apoptotic phenotype of FLS, promoting their death [79]. Studies found that resveratrol inhibits autophagy by downregulating Beclin‐1 and LC3A/B, promoting mitochondrial‐dependent apoptosis and alleviating AA [80]. PADI4 gene silencing inhibits autophagy and induces FLS apoptosis, indicating the potential value of epigenetic regulation [81].

Autophagy counteracts apoptosis in RA through multiple mechanisms. TNF‐α enhances autophagic activity by activating the PI3K/AKT/mTOR pathway, inhibiting FLS apoptosis, and forming an “inflammation‐autophagy‐survival” vicious cycle. FLS enhance resistance to MTX by increasing the expression of HMGB1 and Beclin‐1, inducing autophagosome formation [82]. TNF‐α, IL‐1β, or LPS can stimulate the upregulation of protein tyrosine phosphatase non‐receptor type 2 (PTPN2), which promotes FLS survival by inhibiting apoptosis and enhancing autophagy [83]. Upregulating DNM1L inhibits apoptosis and activates NF‐κB inflammatory signaling by increasing LC3B expression and ROS levels [84].

The interaction between autophagy and apoptosis in RA is complex and diverse; a deeper understanding of the interaction between autophagy and apoptosis could provide new targets and strategies for clinical treatment.

### 5.2 Interaction Between Necroptosis and Autophagy

In RA, the abnormal regulation of necroptosis and autophagy directly participates in the occurrence and progression of the disease. Studies have found that upregulated LC3B and Beclin‐1 significantly inhibit RIPK1/RIPK3 activation and promote the abnormal survival and invasive ability of RA FLS [85]. Inflammatory factors such as TNF‐α activate necroptotic pathways in FLS, leading to cell death and the release of inflammatory mediators, further exacerbating joint inflammation [86]. Notably, TNF‐α also activates NF‐κB signaling, which enhances autophagic activity and inhibits caspase‐8 function, forming an “autophagy‐necroptosis resistance” vicious cycle.

On the other hand, autophagic dysfunction may lead to the accumulation of damaged organelles and misfolded proteins, further promoting necroptosis. This dynamic imbalance is especially evident in clinical treatments: studies on MTX resistance suggest that FLS accelerate MTX degradation via upregulated autophagy, simultaneously reducing the release of necroptosis‐related factor HMGB1, leading to drug failure [87]. In contrast, commonly used biologics, such as anti‐TNF‐α therapy, may indirectly suppress necroptosis pathways by excessively enhancing autophagic activity, weakening the inflammation‐clearing effects.

Therefore, the regulatory mechanisms between necroptosis and autophagy are very complex. Animal experiments provide important evidence: in a CIA mouse model, co‐administration of autophagy inhibitors (eg., chloroquine, as described in 4.5) and necroptosis inducers (TSQ) significantly reduced joint destruction [88]. Clinical sample analysis also showed that the autophagy marker LC3B co‐expressed with the necroptosis inhibitor cFLIP in RA synovial tissue, further suggesting the close interaction between the two pathways [66]. Future research should further explore the key regulatory factors of these pathways to provide new therapeutic approaches for RA.

### 5.3 Interaction Between Apoptosis and Necroptosis

Furthermore, during the progression of RA, there is an interrelationship between apoptosis and necroptosis. Studies suggest that SMAC mimetics (SMs) inhibit the anti‐apoptotic effects of SMAC by competing with CIAP1/2 proteins, thereby enhancing TNF‐α release and activating RIPK1, RIPK3, and MLKL in pro‐inflammatory M1 macrophages, thereby inducing apoptosis and necroptosis [89].

Additionally, compared to traditional TNF‐α inhibitors like etanercept, geldanamycin regulates cell death balance through a dual mechanism: on one hand, it inhibits RIPK1 kinase activity and NF‐κB inflammatory pathways, blocking TNF‐α‐induced necroptotic signals; on the other hand, it enhances caspase‐8 activation, promoting apoptosis in RA synovial cells. In a CIA mouse model, this drug significantly reduced synovial hyperplasia and improved arthritis phenotypes, showing better clinical potential than etanercept [90].

### 5.4 Interaction Between Apoptosis and Pyroptosis

The process of cell death is typically initiated by mitochondrial release of apoptotic factors. When cells are subjected to TNF and oxidative stress, mitochondria release a series of pro‐apoptotic factors, ultimately leading to apoptosis. However, in certain cases, especially when caspase‐8 activity is inhibited, cells may switch to necroptosis. The presence of GSDME protein has been found to promote the shift from apoptosis to pyroptosis, further increasing the diversity of cell death pathways. Moreover, lactoferrin and GSDMD participate in inducing NETosis to digest pathogens, highlighting the interaction between pyroptosis and NETosis pathways [91].

Additionally, inflammasomes are related to pyroptosis, and there is a complex interaction between apoptosis and pyroptosis [25]. Caspase‐8 plays a key regulatory role in cell death pathways as a central regulatory node in the cysteine‐aspartic protease family. The caspase family coordinates the dynamic balance between apoptosis execution and necroptosis inhibition through a complex protein–protein interaction network [92]. The following sections will elaborate on its dual function in regulating cell death.

### 5.5 Interaction Between Apoptosis and Ferroptosis

The presence of GSDME protein can promote the transition from apoptosis to pyroptosis. Similarly, there is a potential conversion between apoptosis and ferroptosis. Iron accumulation regulates osteoblast apoptosis through the XIST/miR‐758‐3p/caspase‐3 axis; ROS play a key role in ferroptosis‐induced osteoblast necroptosis, and the RIPK1/RIPK3/MLKL pathway is the core mechanism of iron overload‐induced necroptosis in vitro [28, 93, 94].

### 5.6 Interaction Between Ferroptosis and Pyroptosis

Moreover, ferroptosis, mediated by iron ions, has a certain connection with pyroptosis. Studies have shown that in iron‐treated MC3T3‐E1 osteoblasts, the accumulation of iron increases ROS production and activates caspase‐3, which further cleaves GSDME protein, causing the transition from TNF‐α‐induced apoptosis to pyroptosis. The GPX4 and GSDMD signals play important roles in triggering ferroptosis and pyroptosis, respectively, which may lead to excessive ferroptosis and pyroptosis in bone cells in RA, causing abnormal bone cell function or death [95]. The interaction between GPX4 and GSDMD signals provides new research directions and potential targets for RA treatment.

### 5.7 Caspase‐8 Acts as a Molecular Hub for Cell Death Modalities

As a core regulator, caspase‐8 coordinates different modes of cell death through the following mechanisms. In apoptosis, upon death receptor activation, FADD‐mediated caspase‐8 activation triggers the downstream caspase‐3/7 cascade, executing the classical apoptotic program [96]. Additionally, when caspase‐8 activity is inhibited (eg., by viral infection or pharmacological intervention), RIPK1/RIPK3 oligomerizes to form the necrosome, activating MLKL‐mediated membrane rupture and potentially shifting the cell toward necroptosis [97].

Caspase‐8 can directly induce pyroptosis by cleaving GSDMD or indirectly promote NLRP3 inflammasome activation via the RIPK3‐MLKL axis, leading to the release of pro‐inflammatory factors such as IL‐1β and triggering pyroptosis [98]. In the regulation of both apoptosis and pyroptosis, caspase‐8 plays a critical role. Upon activation, it cleaves the Pannexin‐1 channel via caspase‐3, inducing potassium efflux and activating the NLRP3 inflammasome. If caspase‐8 remains active, it further activates caspase‐3 to induce apoptosis; if its activity is inhibited, the cell shifts toward caspase‐1 activation and pyroptosis [99].

Furthermore, caspase‐3/8 can directly act on GSDMD family proteins to regulate pyroptosis, while delayed GSDMD pore formation may cause a transition from pyroptosis to apoptosis [100].

The PCD pathways form a dynamic interactive network centered on caspase‐8. The balance among these pathways is regulated by microenvironmental signals and metabolic states. Deciphering these mechanisms provides new insights for developing targeted therapeutic strategies.

## 6 Clinical Implications and Translational Perspectives

As reviewed in this article, the dysregulation of multiple PCD pathways in RA FLS extends beyond elucidating pathogenic mechanisms, offering substantial translational potential for refining RA management.

These insights provide promising avenues for novel therapeutic development. Key molecular nodes within specific PCD pathways—such as RIPK1/RIPK3/MLKL in necroptosis, NLRP3/GSDMD in pyroptosis, GPX4/xCT in ferroptosis, PAD4 in NETosis, and copper homeostasis regulators—represent compelling targets for next‐generation therapies. Understanding the mechanisms underlying apoptosis resistance (eg., p53 inactivation, endoplasmic reticulum stress tolerance, survival pathway activation) and PCD crosstalk informs strategies to overcome drug resistance and enables the design of precision combination therapies. Nevertheless, robust clinical evidence remains limited at present; these therapeutic prospects are largely extrapolated from preclinical/basic studies and require further validation.

Secondly, PCD dysregulation serves as a rich source for biomarker discovery and validation. Molecules associated with specific PCD modes—such as soluble RIPK1/MLKL, PTX3, GSDMD‐N‐terminal fragments, NETosis derivatives (MPO–DNA complexes, calprotectin), indices of iron metabolism (ferritin, transferrin saturation), copper and copper‐binding proteins (ceruloplasmin), and lipid peroxidation products (MDA, 4‐HNE)—detected in serum or synovial fluid hold promise for improving RA diagnosis, stratifying disease subtypes, assessing disease activity, predicting responses to targeted therapies, and monitoring treatment efficacy and prognosis. At present, there are no broadly validated, reliable panels for routine clinical use; most signals derive from exploratory cohorts and will need prospective, standardized studies.

Building on the above biomarker and pipeline landscape, current and emerging interventions can be reinterpreted through the lens of PCD modulation, informing rational combinations and patient endotyping. For instance, the efficacy of sulfasalazine may be partially attributable to modulation of ferroptosis susceptibility, while biologics (anti‐TNFα, anti‐IL‐6R) likely exert effects partly by normalizing dysregulated PCD in FLS (eg., suppressing necroptosis/pyroptosis). The anti‐RA actions of natural compounds (eg., triptolide, icariin, resveratrol, quercetin) are increasingly linked to their regulation of multiple PCD pathways, providing a mechanistic basis for their use or derivative development. While challenges remain regarding target specificity, pathway complexity, and prospective biomarker validation, strategically targeting dysregulated PCD pathways and deploying associated biomarkers offers substantial promise for advancing personalized and more effective therapeutic strategies in RA.

## 7 Conclusions and Future Directions

RA is a complex multifactorial disease involving intricate interactions between various immune cells and cell death pathways. The pathological process of RA includes not only the excessive proliferation of synovial cells and the infiltration of inflammatory cells but also the activation of various forms of PCD. These forms of PCD include apoptosis, pyroptosis, necroptosis, and ferroptosis, which are not isolated events but interconnected and regulated processes that collectively drive the pathogenesis of RA.

Although significant progress has been made in RA treatment, a comprehensive understanding of its complex pathological mechanisms remains to be achieved. Future research should explore the specific roles of different PCD pathways in RA and their interconnections, especially how they collectively regulate inflammatory responses and immune balance. Moreover, developing targeted therapies for emerging drug targets will be an important direction in RA treatment research. By integrating studies on these cell death pathways, new strategies for the diagnosis, treatment, and prevention of RA can be developed.

## Author Contributions

**Xiaorong Zhi:** conceptualization, methodology, writing – original draft, visualization. **Hong Zhu:** conceptualization, writing – review and editing. **Xiaoyan Sun:** writing – review and editing. **Zhaoxia Wang:** writing – review and editing. **Lin Wang:** conceptualization, writing – original draft, writing – review and editing, supervision, management, and coordination responsibility for the research activity planning and execution. **Guanhua Song:** conceptualization, methodology, writing – original draft, writing – review and editing, supervision, management, and coordination responsibility for the research activity planning and execution.

## Conflicts of Interest

The authors declare no conflicts of interest.

## Data Availability

The authors have nothing to report.
